# Evolving Patterns of TPO-RA Use in Children: A Decade of Single-Centre Experience and Narrative Review

**DOI:** 10.3390/ijms27125175

**Published:** 2026-06-07

**Authors:** Bartosz Urbański, Małgorzata Gaszyńska, Izabela Kasprzycka, Olga Kowalczyk, Olga Wegner, Magdalena Wojdalska, Monika Zjawiona, Wojciech Młynarski, Szymon Janczar

**Affiliations:** Department of Pediatrics, Oncology and Hematology, Medical University of Lodz, 251 Pomorska Street, 92-213 Lodz, Poland

**Keywords:** platelets, thrombopoietin receptor agonists, immune thrombocytopenia, aplastic anemia, inherited platelet disorders

## Abstract

Thrombopoietin receptor agonists (TPO-RAs) have become an important component of pediatric immune thrombocytopenia (ITP) management, with growing evidence supporting their use in other hematologic disorders. We conducted a retrospective single-center study evaluating TPO-RA use at a pediatric oncohematology center in Poland between 1 January 2016, and 31 March 2026. Clinical indications, treatment patterns, efficacy, and safety outcomes were analyzed. Treatment response was defined as initial response (IR, platelet count [PLT] > 50 × 10^9^/L), complete response (CR, PLT > 100 × 10^9^/L), and sustained response (SR, PLT > 50 × 10^9^/L maintained in ≥75% of visits over 6 months). Thirty-five patients were included in the analysis. The most common indication was ITP (27/35, 77%), followed by severe aplastic anemia (5/35, 14%), poor graft function (2/35, 6%), and inherited platelet disorders (1/35, 3%). TPO-RA use increased over time, with more than half of patients (20/35, 57%) initiating therapy between 2024 and 2026. Overall IR, CR, and SR rates were 77%, 63%, and 61%, respectively, with a median time to response of 7 days (interquartile range [IQR], 7–12.5). TPO-RAs were increasingly used earlier in the disease course, including in rescue and off-label settings. Treatment was well tolerated, with only grade 1–2 adverse events reported. Our real-world data confirm the high efficacy and favorable safety profile of TPO-RAs in pediatric patients and support their expanding role in pediatric hematology beyond disease-specific indications.

## 1. Introduction

Thrombopoietin (TPO) is a growth factor critical for the regulation of platelet production. The existence of a then-unknown circulating mediator controlling thrombopoiesis had been hypothesized as early as the 1950s. A breakthrough came in 1992, when French researchers reported cloning the human homolog of the *v-mpl*, an oncogene derived from myeloproliferative leukemia virus, which was subsequently designated c-MPL [1,2]. Shortly thereafter, independent groups identified the c-MPL ligand with potent thrombopoietic activity, later termed TPO [3,4,5].

**Figure 1:**
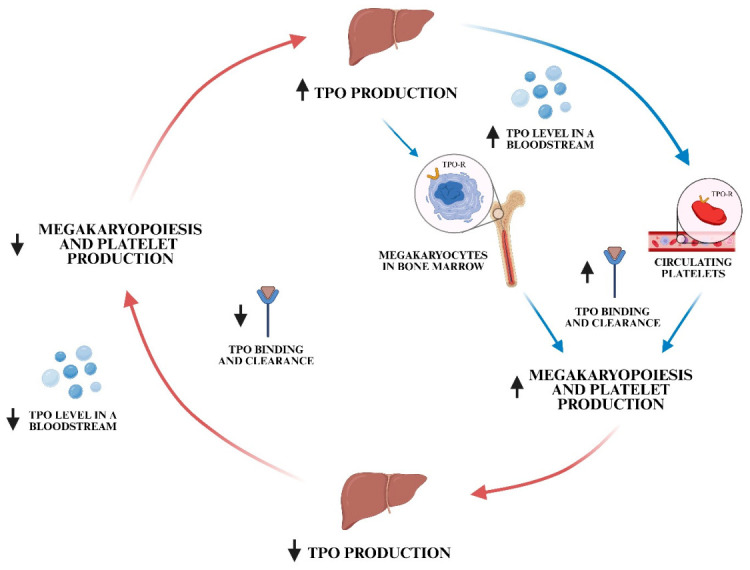
Schematic representation of the feedback regulation of thrombopoietin (TPO) levels. Liver-derived TPO is constitutively produced and cleared through binding to c-MPL receptors expressed on circulating platelets and bone marrow megakaryocytes, thereby establishing a negative feedback loop that regulates platelet production. Blue arrows indicate stimulatory effects, whereas red arrows denote inhibitory pathways. Abbreviations: TPO, thrombopoietin.

This 332-amino-acid glycoprotein is produced predominantly by the liver, and its plasma concentration is generally inversely proportional to platelet count and megakaryocyte mass. In the classical model, circulating thrombocytes expressing c-MPL bind free TPO, internalize it, and subsequently mediate its degradation. Accordingly, an increased platelet mass reduces circulating TPO levels, whereas thrombocytopenia decreases hormone clearance, leading to elevated plasma TPO concentrations [6]. However, studies on the pathogenesis of immune thrombocytopenia (ITP) have revealed that TPO levels are often lower than expected for the degree of thrombocytopenia, leading to the concept of its relative deficiency. According to this model, the increased megakaryocyte mass observed in ITP, which likewise expresses c-MPL, contributes substantially to TPO clearance, resulting in lower circulating TPO concentrations (Figure 1) [7]. Consequently, these observations prompted a strong rationale for the therapeutic exploitation of this pathway in thrombocytopenia [8].

The first generation of TPO receptor agonists included recombinant human thrombopoietin (rhTPO) and pegylated recombinant human megakaryocyte growth and development factor (PEG-rHuMGDF). Despite initially promising results, the clinical development of these agents was limited by the generation of cross-reactive antibodies, which eventually led to therapy refractoriness and severe thrombocytopenia [9,10]. This unexpected toxicity redirected subsequent research toward molecules that lack structural homology to endogenous TPO.

Romiplostim, a subcutaneously administered c-MPL-binding peptibody, demonstrated rapid clinical responses in early clinical trials. Phase II studies in patients with chronic ITP showed dose-dependent increases in platelet counts during short-term therapy, without significant safety concerns or development of neutralizing antibodies [11,12]. These findings were confirmed in two randomized, double-blind phase III trials, in which romiplostim was administered for up to 24 weeks and resulted in sustained platelet responses compared with placebo in both splenectomized and non-splenectomized patients [13].

In parallel, eltrombopag, an oral small-molecule c-MPL agonist, demonstrated early responses in over 80% of patients with chronic ITP in a phase II study [14]. These promising results were subsequently corroborated in the RAISE trial, a randomized phase III study demonstrating improved platelet responses and reduced bleeding risk in patients receiving eltrombopag compared with placebo [15].

Following these positive clinical data, in 2008, romiplostim and eltrombopag received approval from the U.S. Food and Drug Administration (FDA) for the treatment of adults with chronic ITP who had an inadequate response to corticosteroids, immunoglobulins, or splenectomy [16,17]. The robust efficacy of TPO-RAs observed in adults prompted the initiation of clinical trials in children, including the PETIT and PETIT2 studies [18,19]. With the favorable results of these trials leading to the extension of indications to the pediatric population (eltrombopag in 2015 and romiplostim in 2018), as well as the approval of additional agents, avatrombopag and lusutrombopag, TPO-RAs have revolutionized the management of thrombocytopenia. Growing evidence and clinical experience have made TPO-RAs an increasingly important part of ITP treatment, with a gradual shift toward earlier use, including first-line therapy in selected patients [20,21]. Moreover, subsequent studies established eltrombopag as an effective adjunctive therapy in severe aplastic anemia (SAA) and expanded the rationale for broader off-label use of TPO-RAs in post-transplant thrombocytopenia, chemotherapy- and immunotherapy-induced thrombocytopenia, as well as inherited platelet disorders (IPDs) [22,23,24,25].

Almost a decade after the introduction of TPO-RAs into pediatric clinical practice, we aimed to evaluate evolving patterns of their use, treatment efficacy, and safety at a single pediatric oncohematology center in Poland.

## 2. Results

### 2.1. Cohort Overview

The study cohort comprised 35 patients, with a slight male predominance (male-to-female ratio, 1.19). The median age at diagnosis was 10.36 years (interquartile range [IQR], 5.49–13.89). TPO-RA therapy was initiated at 10.78 years (IQR, 6.8–14.65), following a median interval of 231 days from diagnosis to treatment initiation (IQR, 90–424). Median follow-up duration from TPO-RA initiation to the last available clinical contact was 621 days (IQR, 287–1005). Notably, 8 patients (23%), mostly among those starting treatment between 2024 and 2026, received TPO-RAs as rescue therapy. Overall, TPO-RA use increased markedly over time, with more than half of initiations occurring during the most recent period (2024–2026: 20/35, 57%).

**Figure 2:**
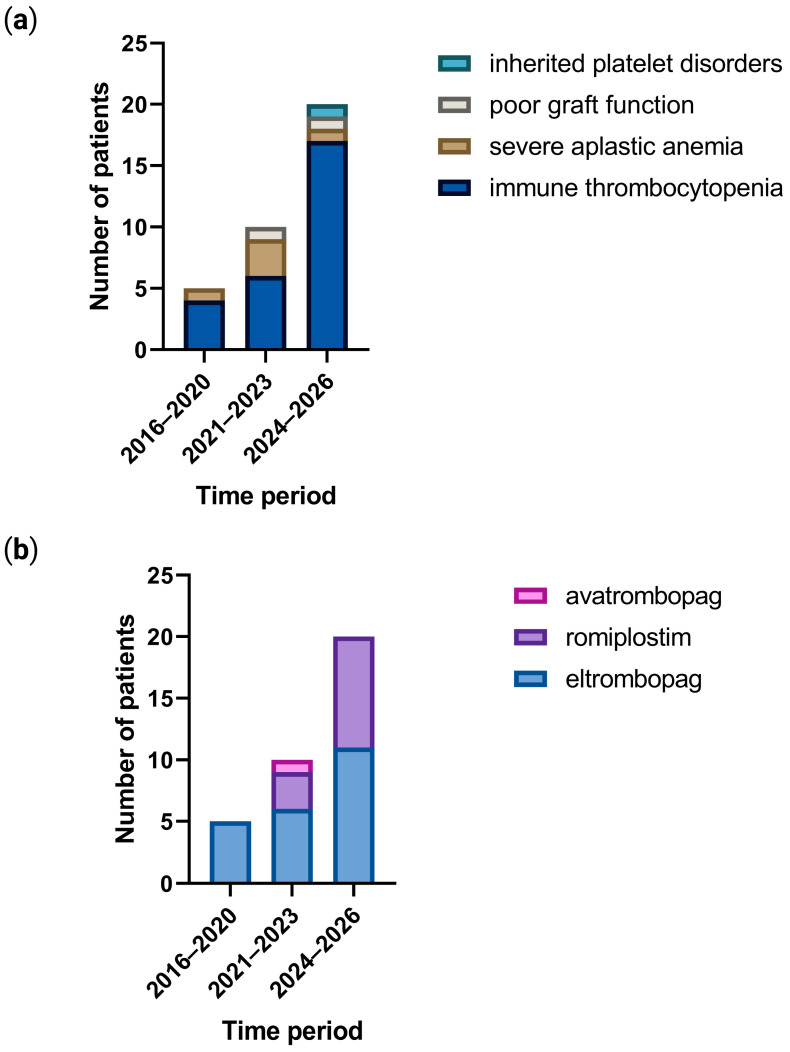
Temporal trends in thrombopoietin receptor agonist (TPO-RA) use. (**a**) Clinical indications for TPO-RA initiation across successive time periods. (**b**) Selection of first-line TPO-RA agents over time. Bars represent the number of patients initiating treatment within each interval.

The predominant indication was ITP (27/35, 77%), followed by SAA (5/35, 14%), poor graft function (PGF; 2/35, 6%), and IPDs (1/35, 3%). No patients received TPO-RAs for chemotherapy-related thrombocytopenia. Across successive time intervals, increasing ITP-related use was observed, accompanied by the emergence of new indications, including SAA, PGF, and IPDs. Eltrombopag was the most commonly used first-line TPO-RA (22/35, 63%), followed by romiplostim (12/35, 34%) and avatrombopag (1/35, 3%). Agent selection became more diverse over time, with a relative increase in romiplostim use, particularly in ITP. Trends in indications and TPO-RA selection over time are illustrated in Figure 2.

The overall initial response (IR1) rate to the first TPO-RA was 77%, with a median time to response of 7 days (IQR, 7–12.5), consistent with rapid platelet recovery among responders. IR1 rates varied across agents, reaching 100% with romiplostim and avatrombopag, compared with 64% with eltrombopag. Complete response (CR1) was observed in 63% of patients overall, with marked differences between agents: 92% for romiplostim, 100% for avatrombopag, and 45% for eltrombopag. Sustained response (SR1) was achieved in 61% of evaluable patients, with comparable rates between eltrombopag (57%) and romiplostim (67%).

Regarding the first TPO-RA exposure, therapy remained ongoing and was discontinued in an equal proportion of patients (14/34 each, 41%), whereas switching to an alternative agent was required in 6/34 patients (18%). Treatment status was not assessable in one patient (P14 in Supplementary Table S1) because of early loss to follow-up after transition to adult care. Disease remission was the most common reason for first-therapy discontinuation, whereas lack of response represented the leading indication for switching.

At the last follow-up, overall TPO-RA therapy remained ongoing in 20/34 patients (59%), whereas treatment had been discontinued in 14/34 patients (41%). Disease remission in patients with ITP was the most common reason for overall treatment discontinuation (5/14, 36%). Four of these five patients achieved sustained complete response off treatment, according to the definition proposed by Guillet et al. (PLT > 100 × 10^9^/L and absence of bleeding over a 52-week follow-up period) [26]. Treatment was discontinued due to lack of efficacy in only 3 patients. Importantly, no severe hemorrhagic events were observed during TPO-RA therapy. Mild bleeding episodes occurred in 7 patients (20%), most commonly epistaxis (57% of bleeding events), followed by mucosal bleeding (29%) and menorrhagia (14%).

Detailed treatment characteristics and outcomes are summarized in Table 1.

Treatment-related adverse events (AEs) were reported in 9/35 patients (26%). All events were mild (CTCAE v5.0 grade 1–2) and did not require therapy discontinuation [27]. The most frequent events included hepatotoxicity and hematologic abnormalities, mostly associated with eltrombopag. Detailed characteristics of TPO-RA-related AEs are summarized in Table 2, whereas patient-level data are provided in Supplementary Table S1.

Detailed patient-level characteristics of the study cohort are provided in Supplementary Table S1.

### 2.2. Immune Thrombocytopenia (ITP)

Among the study cohort, the vast majority of patients (27/35, 77%) had a diagnosis of ITP. Eltrombopag was the most commonly used first-line TPO-RA (15/27, 55%), although during the most recent period its use became comparable to romiplostim (11 vs. 9 patients; Figure 2). Romiplostim appeared to be the only initial agent used in patients younger than 5 years. However, age distributions were otherwise comparable between treatment groups.

The overall IR1 and CR1 in patients with ITP were 85% and 70%, respectively. Higher rates were observed with romiplostim (IR1 100%, CR1 91%) and avatrombopag (IR1 100%, CR1 100%) than with eltrombopag (IR1 73%, CR1 53%), although the difference in IR1 rates did not reach statistical significance (*p* = 0.11). Notably, SR1 was achieved by 71% of evaluable patients, with similar outcomes for eltrombopag (73%) and romiplostim (63%; *p* = 0.66).

Switching between TPO-RA agents was required in 6/27 patients (22%), most commonly from eltrombopag to romiplostim. Following therapy modification, IR2 and CR2 were each achieved in 33% of patients, while SR2 was observed in 17% of cases. Detailed treatment outcomes and switching patterns are presented in Table 3.

During follow-up, several additional treatment modifications were observed, including re-exposure to the initial TPO-RA or transition to a third-line agent, with all patients achieving at least an initial response. Remission in a subset of patients with refractory ITP was achieved only after combining TPO-RA therapy with immunosuppressive agents, including corticosteroids, rituximab (RTX), or mycophenolate mofetil (MMF). Of particular interest, two patients received concomitant administration of eltrombopag and romiplostim as rescue therapy for refractory ITP complicated by severe bleeding, and both achieved clinical response [28].

### 2.3. Severe Aplastic Anemia (SAA)

Eltrombopag was the only TPO-RA used in patients with SAA (*n* = 5). All patients received eltrombopag in combination with immunosuppressive therapy, most commonly corticosteroids and cyclosporine, according to EWOG-SAA protocols [29]. TPO-RA use in SAA is not standard practice in Poland and was implemented on an individual basis, primarily because of inadequate response to immunosuppression. Overall, IR and CR were achieved in 40% (2/5) and 20% (1/5) of patients, respectively, whereas no SRs were observed. Most patients (4/5) ultimately proceeded to hematopoietic stem cell transplantation (HSCT), which represented the definitive treatment approach in this subgroup. During follow-up, one patient (P7, Supplementary Table S1) developed a paroxysmal nocturnal hemoglobinuria clone approximately three months after SAA diagnosis. Eltrombopag was eventually discontinued after six months because of lack of efficacy. Several months later, follow-up bone marrow evaluation demonstrated multilineage dysplasia, 3–4% blasts, and newly detected monosomy 7, consistent with progression to myelodysplastic syndrome. Subsequent reassessment revealed further evolution with 20–30% B-lineage lymphoblasts, leading to transformation to acute lymphoblastic leukemia. Following achievement of remission, the patient underwent HSCT from a related donor and has remained in sustained remission for more than three years.

### 2.4. Poor Graft Function (PGF)

During the study period, TPO-RA (eltrombopag) was administered in two cases of PGF following HSCT. One patient achieved clinical response (IR, CR, and SR), although concomitant immunosuppressive and cytotoxic therapies limited objective assessment of treatment efficacy. Both patients ultimately died due to progression of the underlying disease.

### 2.5. Inherited Platelet Disorders (IPDs)

**Figure 3:**
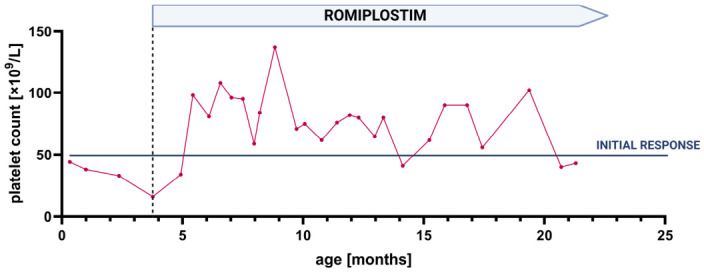
Platelet count dynamics and romiplostim treatment timeline in a patient with Wiskott–Aldrich syndrome, illustrating individualized off-label thrombopoietin receptor agonist use in a rare inherited platelet disorder. The pink line with dots represents serial platelet count measurements over time. The horizontal line denotes the initial response threshold (PLT > 50 × 10^9^/L). Definitions of response endpoints are provided in the Section 4.

Of particular interest, TPO-RA was also administered to an infant diagnosed with IPD. The patient presented with severe thrombocytopenia and bleeding symptoms since birth. Due to the lack of response to standard ITP-directed therapy, molecular testing was performed and identified a pathogenic variant in the *WAS* gene (c.134C>T; p.Thr45Met), consistent with Wiskott–Aldrich syndrome (WAS). Given isolated severe thrombocytopenia, prior gastrointestinal bleeding, and emerging evidence suggesting potential TPO-RA efficacy in patients with WAS, off-label romiplostim was initiated [30]. Rapid platelet count recovery, accompanied by improvement in functional tests (reflected by shortened PFA-200 closure times), was observed. Moreover, no severe bleeding episodes or significant treatment-related AEs were observed during 18 months of follow-up. The patient is currently undergoing evaluation for allogeneic HSCT. Longitudinal platelet count dynamics and treatment course are illustrated in Figure 3.

## 3. Discussion

### 3.1. General Considerations and Mechanistic Context

In this single-center retrospective study, we analyzed our experience with TPO-RAs in a pediatric hematology setting, focusing on their efficacy across an expanding spectrum of indications and evolving patterns of clinical use. Over the past decade, TPO-RAs have become a cornerstone of pediatric ITP management, supported by robust evidence demonstrating high efficacy, favorable safety, and good tolerability. In parallel, their clinical use has extended beyond ITP to include conditions such as SAA, PGF, and IPDs [22,23,24,25]. Our findings align with these evolving trends, demonstrating not only increasing utilization of TPO-RAs over time but also a gradual broadening of clinical indications and changing treatment patterns. Although ITP remained the predominant indication for TPO-RA initiation throughout all analyzed periods, a progressively broader spectrum of diagnoses emerged in subsequent years, including individualized off-label applications. Despite this heterogeneity, the overall IR rate in the entire cohort reached 77%. Importantly, bleeding events during TPO-RA therapy occurred in only 20% of patients and were uniformly mild, further supporting the favorable clinical effectiveness of these agents. The comparatively lower efficacy of eltrombopag, reflected by IR and CR rates in the overall cohort, may partly relate to its exclusive use in more complex or less established indications, such as SAA or PGF. Nevertheless, SR rates remained comparable across different TPO-RAs, suggesting that long-term effectiveness may depend less on the specific agent and more on patient- and disease-specific factors.

The approval of new TPO-RAs for pediatric use has gradually diversified therapeutic options. Nevertheless, eltrombopag and romiplostim remain the most frequently used agents in our cohort. This is largely driven by their greater availability, in contrast to avatrombopag, which was only approved for pediatric use in 2025 [31]. In Poland, both eltrombopag and romiplostim have been available for several years under a dedicated drug program for pediatric patients with ITP lasting more than six months and insufficient response to prior therapies, which has undoubtedly influenced the selection of TPO-RAs in routine clinical practice.

Importantly, in our cohort, particularly among patients with ITP, we observed a notable shift toward more rapid TPO-RA initiation in recent years, in some cases within the first week after diagnosis. This approach was particularly evident in patients presenting with severe or life-threatening bleeding. In this subgroup, IR was achieved in 75% of patients, suggesting that prompt administration of TPO-RAs may provide rapid and clinically meaningful platelet recovery even in high-risk scenarios. The increasing use of TPO-RAs earlier in the disease course likely reflects evolving treatment paradigms, growing clinical experience, drug availability, and institutional preferences. While these observations should be interpreted with caution due to the retrospective nature of the study, they support the growing body of evidence advocating for a more flexible and earlier incorporation of TPO-RAs into treatment algorithms [32].

In our cohort, TPO-RA therapy was generally well tolerated, with AEs observed in 26% of patients. All of them were mild (CTCAE grade 1–2), reversible, and none required treatment discontinuation. Similar findings have been reported in both pre-approval clinical trials and the later EXTEND study, the largest long-term evaluation of eltrombopag safety [11,12,13,15,33]. However, it is possible that some AEs in our analysis were underreported, particularly headache and upper respiratory tract infections, as most events were collected retrospectively from routine clinical documentation. On the other hand, eltrombopag-related hepatobiliary toxicity and mild hematologic abnormalities were relatively frequent (>10%) compared with existing literature, reflecting the known effects of TPO-RAs on other hematopoietic lineages [18,19]. In contrast to some adult cohorts, we identified only a single case of bone marrow fibrosis. The affected patient (P3 in Supplementary Table S1) had chronic refractory ITP after multiple prior therapeutic interventions including sequential exposure to three TPO-RAs. Baseline marrow assessment before romiplostim initiation showed no evidence of increased fibrosis. Subsequent clinically indicated biopsies identified moderate reticulin fibrosis, without clonal abnormalities or features suggestive of progression beyond ITP. Notably, repeat bone marrow evaluation performed before transition to adult care showed partial regression of these changes. Of note, routine trephine biopsy is not standard practice in pediatric ITP, which may have limited the accurate assessment of this AE in our analysis. Moreover, no thromboembolic events were observed, although such events have been sporadically reported in long-term follow-up studies [33,34,35]. Notably, the overall risk of thrombosis appears to be influenced primarily by additional patient-related risk factors, particularly in adult populations.

To provide a biological framework for interpreting these observations across different indications, the mechanistic properties of currently available TPO-RAs warrant brief consideration. All currently available TPO-RA agents exert their effects through activation of the c-MPL receptor, expressed primarily on megakaryocyte progenitors and platelets. Receptor activation stimulates canonical signaling pathways, including JAK2/STAT5, PI3K/AKT, MAPK, and MEK/ERK cascades, ultimately promoting megakaryocyte proliferation, differentiation, and platelet production [36,37,38]. Despite this shared functional endpoint, currently available agents differ substantially in their molecular structure, receptor-binding characteristics, and pharmacokinetic profiles.

Romiplostim is a peptibody administered subcutaneously that binds the extracellular domain of c-MPL and competes with endogenous TPO. By contrast, eltrombopag, avatrombopag, lusutrombopag, and hetrombopag are orally given non-peptide molecules that interact with the transmembrane domain at a site distinct from the endogenous ligand-binding region [38,39]. This mechanism enables non-competitive receptor activation and may permit additive or synergistic interactions with endogenous TPO. This feature may partly explain the efficacy of eltrombopag in settings of inflammation-related impairment of TPO signaling, such as SAA and inherited bone marrow failure syndromes (IBMFS).

Moreover, data from preclinical in vitro studies indicate that individual TPO-RAs may differ in their activity across stages of megakaryopoiesis. Romiplostim appears to act predominantly on mature megakaryocytes, whereas eltrombopag exerts broader effects at earlier stages of hematopoiesis, including progenitors [40,41]. This distinction may contribute to eltrombopag efficacy in SAA, in which expansion of multipotent progenitor cells is critical for hematologic recovery [42]. Collectively, these mechanistic differences may translate into distinct clinical response patterns and provide a biological rationale for individualized strategies, including agent switching or, in selected circumstances, concomitant use.

More recent data suggest that, beyond their stimulatory role in megakaryopoiesis, TPO-RAs may exert immunomodulatory effects, particularly romiplostim. Studies in chronic ITP have demonstrated increases in regulatory T cells (Tregs) accompanied by downregulation of autoreactive T lymphocytes during TPO-RA therapy. A central role in the induction of immune tolerance has been attributed to transforming growth factor beta (TGF-β), a key cytokine within the TGF-β–Treg axis that promotes Treg differentiation and limits autoimmunity. Its levels have been shown to increase in parallel with sustained response [43,44]. In another study, Schifferli et al. proposed that the Fc fragment of romiplostim may interact with Fcγ receptors on antigen-presenting cells, thereby modulating antigen processing and presentation [45]. It is highly plausible that TPO-RAs’ immunomodulatory activity substantially contributes to durable remission in ITP beyond their primary role in stimulating platelet production.

In addition to its thrombopoietic effects, eltrombopag demonstrates several pleiotropic biological activities that may further influence clinical outcomes. In leukemic cell lines, eltrombopag exhibited potent iron-chelating activity, leading to depletion of the intracellular iron pool. This reduction was associated with decreased reactive oxygen species production, resulting in an antiproliferative effect on abnormal or stressed hematopoietic clones, while preserving or even promoting the expansion of normal progenitor cells [46,47,48]. This mechanism is thought to be particularly relevant in SAA, in which oxidative damage and immune-mediated destruction contribute to stem cell depletion [49]. Additional preclinical studies indicate that eltrombopag, similarly to endogenous TPO, may enhance non-homologous end joining DNA repair pathways in hematopoietic stem cells, potentially contributing to genomic stability in selected IBMFS, including Fanconi anemia [50,51]. Emerging reports also suggest additional effects involving mesenchymal stromal cells, platelet function, and antimicrobial responses [52,53,54]. Further research is needed to better define the full spectrum of TPO-RA activity and its clinical implications.

**Figure 4:**
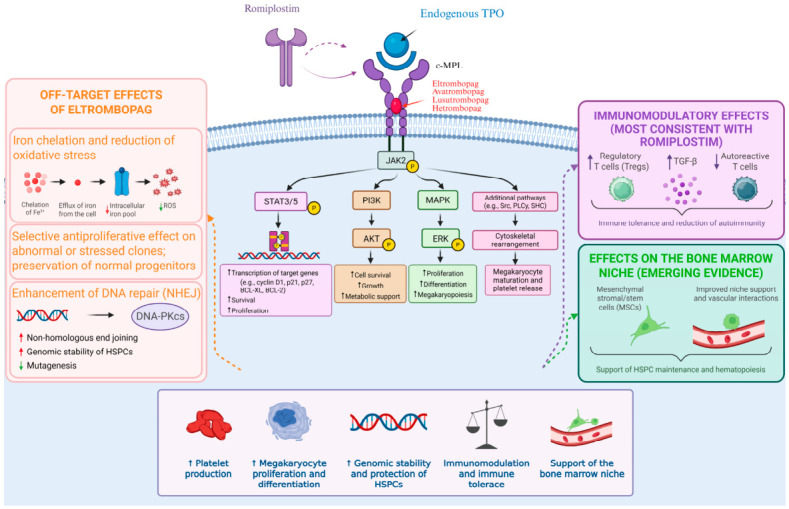
Schematic representation integrating both canonical c-MPL–mediated signaling pathways and emerging non-canonical effects of thrombopoietin receptor agonists, reflecting the current understanding of their multifaceted biological activity [6,36,44,47,50,52]. Abbreviations: TPO, thrombopoietin; c-MPL, thrombopoietin receptor; JAK2, Janus kinase 2; STAT, signal transducer and activator of transcription; PI3K, phosphoinositide 3-kinase; AKT, protein kinase B; MAPK, mitogen-activated protein kinase; ERK, extracellular signal-regulated kinase; ROS, reactive oxygen species; NHEJ, non-homologous end joining; HSPCs, hematopoietic stem and progenitor cells.

Figure 4 summarizes the known cellular mechanisms of TPO-RA action, whereas Table 4 provides an overview of their biochemical and clinical properties as well as approved indications.

To further contextualize our findings, in the following subsections, we discuss treatment patterns and outcomes across study subgroups and compare them with evidence from published clinical trials and real-world data.

### 3.2. Immune Thrombocytopenia (ITP)

With the increasing use of TPO-RAs over time, ITP remained the predominant indication for their initiation, reflecting the expanding and increasingly established role of these agents in clinical practice. In this subgroup, outcomes were favorable (IR1 85%, CR1 70%, SR1 71%) and broadly consistent with available literature, in which platelet responses rates typically range between 50–90% [36]. However, direct comparisons remain challenging due to variability in response definitions across studies. Notably, in the pediatric PETIT and PETIT2 trials, which applied response criteria broadly comparable to IR in our cohort, response rates reached 62% and 80%, respectively [18,19]. Similarly, pediatric romiplostim studies have used heterogeneous endpoint definitions, with response rates spanning from 52% to 88% [58,59,60].

Interestingly, romiplostim demonstrated numerically higher efficacy than eltrombopag in our ITP subgroup, although differences in response outcomes did not reach statistical significance. Although this trend is consistent with findings from the meta-analysis by Zhang et al., suggesting a potential advantage of romiplostim in pediatric ITP, current evidence remains insufficient to clearly establish the superiority of one agent over another [61,62]. Practical considerations therefore appear to play a major role in first-line TPO-RA selection. According to our analysis, in younger children, subcutaneous romiplostim may be preferred due to challenges with oral administration and treatment adherence. On the other hand, oral eltrombopag is often considered more convenient in adolescents, despite the need for dietary restrictions. Conversely, romiplostim allows for flexible dose titration and once-weekly administration, although it necessitates repeated injections that may be less acceptable to some patients and caregivers. As a result, TPO-RA agent selection is largely influenced by a balance between route of administration, expected adherence, and family preference. Emerging oral TPO-RAs, such as avatrombopag, lusutrombopag, and hetrombopag, which lack clinically significant food interactions, may provide a more convenient alternative and shape future treatment patterns in pediatric ITP.

An increasingly observed practice in clinical settings is switching between TPO-RAs. In our cohort, therapy changes most commonly occurred from eltrombopag to romiplostim, reflecting a somewhat lower IR1 for the former agent. In contrast, switching from romiplostim was primarily driven by fluctuating platelet counts and challenges with dose titration. Overall, IR2 and CR2 rates reached 33%, indicating moderate yet clinically meaningful efficacy. Data regarding TPO-RA switching are derived mainly from relatively small clinical trials and real-world data in adults. Response rates generally range from approximately 60% to 80%, with no clear superiority of a specific switching strategy [63,64,65,66,67,68,69]. Pediatric evidence remains sparse and is largely limited to case reports and small observational series [70,71]. Nevertheless, these findings, together with the distinct mechanisms of receptor engagement and additional off-target activities, provide a strong biological rationale for switching strategies in selected clinical settings.

Another important observation from our analysis was the frequent use of a multimodal therapeutic approach. Eight of 27 patients (30%) with ITP received combinations of TPO-RAs and immunosuppressive agents, most commonly RTX or MMF. This strategy appeared to be particularly important for achieving durable remission in selected patients with chronic ITP. Notably, emerging evidence suggests that it may also be justified in selected cases of newly diagnosed refractory disease. Mechanistically, such combinations may be justified by the complementary targeting of impaired platelet production and immune-mediated destruction [72,73]. Most real-world data are derived from adult populations and demonstrate, consistent with our observations, favorable efficacy of combinations involving RTX, MMF, or intensified triple regimens incorporating steroids [74,75,76]. Despite these encouraging results, increasing attention is being paid to the need for careful evaluation of the long-term efficacy and safety of such approaches [77].

By contrast, concomitant administration of two TPO-RAs remains an essentially unexplored therapeutic strategy. In our cohort, we identified two patients who received combined eltrombopag and romiplostim as rescue therapy for refractory ITP, both achieving platelet response. From a mechanistic perspective, this approach may be biologically plausible because of distinct c-MPL receptor binding sites, potentially allowing additive stimulation of thrombopoiesis. However, given the extremely limited clinical experience, further studies are required to adequately assess the efficacy and safety of this strategy.

### 3.3. Severe Aplastic Anemia (SAA)

The role of TPO-RAs in SAA treatment remains more complex and less clearly established than in ITP, particularly in the pediatric population. Although eltrombopag has been approved for use in SAA both in children and adults, available data demonstrate heterogeneous efficacy across different clinical settings and age groups [16].

In adults, substantial clinical trial data indicate that the addition of eltrombopag to standard IST improves hematologic outcomes and accelerates the time to response. The greatest benefit has been observed when eltrombopag is incorporated upfront alongside antithymocyte globulin and cyclosporine, with overall response reaching 70–80%, depending on the study and criteria applied [22,78]. As expected, these outcomes are superior to those reported in earlier studies conducted in refractory settings, in which response rates were closer to 40% [42,79]. Notably, some of these studies also included adolescents aged ≥ 12 years [80].

Pediatric evidence remains comparatively limited, and the results of available studies are often inconsistent. Groarke et al. reported that the addition of eltrombopag to standard IST did not improve response rates compared with a historical pediatric cohort treated with IST alone. Moreover, younger children demonstrated inferior outcomes relative to adolescents [81]. The most comprehensive data derive from the phase II ESCALATE study, which evaluated eltrombopag efficacy in both relapsed/refractory (R/R) settings and as part of an upfront treatment regimen. Overall response at 26 weeks was 54.9%, with somewhat surprising differences between subgroups, reaching 71.4% in R/R patients and 48.6% in treatment-naïve cases. Outcomes in patients aged < 12 years were consistently inferior to those observed in older children across all assessed time points (43.3% vs. 71.4% at week 26). Importantly, this study did not confirm earlier concerns regarding an increased risk of clonal evolution associated with eltrombopag use [82].

In our cohort, eltrombopag was administered exclusively in combination with IST in five patients with SAA, and the median interval from diagnosis to TPO-RA initiation was 90 days (IQR: 86–117). Hematologic responses were limited, with IR achieved in only 40% of patients and CR in 20%, whereas no SRs were observed. Nearly all patients ultimately proceeded to HSCT, which remained the definitive treatment modality in this subgroup. In contrast to published reports, we did not observe clear patterns of response according to patient age, timing of TPO-RA initiation, or therapy duration. However, these observations should be interpreted cautiously given the small sample size and the marked heterogeneity that characterizes pediatric SAA.

Similarly to observations reported in the ESCALATE study, one patient in our cohort developed myelodysplastic syndrome during follow-up, subsequently progressing to leukemia. However, this case should be interpreted with caution, as it likely reflects the intrinsic risk of clonal evolution associated with the natural history of SAA rather than a treatment-related event. Moreover, given the retrospective design of the study, a causal relationship between TPO-RA therapy and leukemic transformation cannot be reliably established.

While eltrombopag remains the only TPO-RA approved for use in SAA, promising results from early studies suggest that romiplostim and avatrombopag may also have therapeutic potential in this setting. Most reports concern patients refractory to standard IST, in whom these agents may represent alternatives, also in cases of resistance or intolerance to eltrombopag [83,84,85,86,87]. For avatrombopag, these observations are consistent with its broad stimulatory effects on hematopoiesis, whereas the efficacy of romiplostim in this setting may appear less intuitive from a mechanistic standpoint. Importantly, the available evidence is derived almost exclusively from adults and should therefore be interpreted with caution in the context of pediatric use.

### 3.4. Poor Graft Function (PGF)

PGF remains a serious complication following HSCT, characterized by persistent cytopenias despite full donor chimerism. Therapeutic options are limited and include supportive care, growth factors, CD34^+^-selected stem cell boosts, and second transplantation, all associated with variable efficacy and significant risks [88,89].

Against this background, TPO-RAs appear to represent a safe, albeit still relatively novel, therapeutic strategy. Current evidence suggests high efficacy of this class of agents, with reported response rates across multiple hematologic lineages exceeding 80% in some analyses [90,91,92,93]. However, most data are derived from small retrospective studies or single-arm clinical trials, limiting the ability to clearly define the role of TPO-RAs in hematologic recovery within this complex clinical setting. Pediatric evidence remains extremely limited and is largely restricted to case reports [94,95].

In our cohort, eltrombopag was administered in two patients with PGF, with hematologic improvement observed in one case. However, the interpretation of treatment efficacy is limited by the concomitant use of other immunosuppressive and cytotoxic therapies, as well as a small study group. Both patients ultimately died due to progression of the underlying disease, highlighting the overall poor prognosis associated with PGF.

Overall, our observations are broadly consistent with published reports suggesting potential efficacy of eltrombopag in PGF. Further research, particularly in pediatric populations, is needed to better define the role, optimal timing, and duration of TPO-RA therapy in this setting.

### 3.5. Inherited Platelet Disorders (IPDs)

IPDs represent a group of rare conditions characterized by quantitative and/or qualitative platelet abnormalities. Advances in molecular diagnostics have substantially expanded their recognized spectrum, which currently encompasses defects involving approximately 80 distinct genes [96]. In many patients, thrombocytopenia is mild and clinically asymptomatic, not requiring therapeutic intervention. Standard management remains largely supportive, relying on platelet transfusions and antifibrinolytic agents to control bleeding or ensure adequate hemostasis before invasive procedures. In selected disorders with severe manifestations, such as WAS or congenital amegakaryocytic thrombocytopenia (CAMT), HSCT remains the curative approach of choice. More recently, gene therapy has also emerged as a therapeutic alternative for specific conditions, including WAS [97,98].

Initial studies investigating the use of TPO-RAs in IPDs focused on short-term eltrombopag administration before surgical procedures in small cohorts of patients with *MYH9*-related thrombocytopenia, demonstrating encouraging hemostatic efficacy [99,100]. These observations provided the basis for exploring broader and longer-term TPO-RAs applications in this setting. Notably, Pecci et al. reported a striking trilineage hematologic response to romiplostim in patients with homozygous *THPO* variants, causing a form of CAMT typically unresponsive to HSCT [101]. Emerging evidence suggests a therapeutic potential of TPO-RAs in WAS-associated thrombocytopenia, with romiplostim response rates reaching up to 70% in some studies [30,102]. However, the most comprehensive cohort to date was reported in a phase II clinical trial by Zaninetti et al., which demonstrated the efficacy of eltrombopag across a heterogeneous group of IPDs, including monoallelic Bernard–Soulier syndrome, *MYH9*-, *ANKRD26*-, *ITGB3*-related thrombocytopenia, and WAS [103].

In our cohort, we described a patient with severe thrombocytopenia within the spectrum of WAS who received off-label romiplostim due to significant bleeding manifestations and emerging supportive evidence from the literature. Treatment resulted in a rapid and sustained platelet response, with no severe bleeding events and good overall tolerability during 18 months of follow-up.

At present, the optimal management of isolated thrombocytopenia in WAS remains highly individualized and depends on disease severity, molecular background, institutional experience, and patient or caregiver preferences. Historically, patients with X-linked thrombocytopenia (a term now considered outdated) were often managed conservatively, with splenectomy performed in cases of severe bleeding. However, accumulating evidence indicates a risk of phenotypic progression over time, including the development of severe immunodeficiency or malignancy. These observations have shifted the therapeutic paradigm toward early consideration of definitive treatment, primarily allogeneic HSCT [97,104,105]. In this context, the use of TPO-RAs, including romiplostim in our patient, should be viewed mainly as a bridging strategy to definitive therapy.

### 3.6. Study Limitations

This study has several limitations that should be considered when interpreting the presented findings. First, this was a retrospective single-centre analysis of a relatively small and clinically heterogeneous cohort across multiple indications and treatment settings, which may limit the generalizability of some observations. In addition, the study reflected real-world clinical practice, including patients with complex clinical characteristics and prior treatment exposure. Several outcomes, including adverse events, were assessed retrospectively from routine medical documentation. Therefore, mild toxicities or subjective symptoms may have been underreported, and standardized patient-reported outcomes and quality-of-life measures were unavailable. Furthermore, although exploratory statistical comparisons were performed in the ITP subgroup, the study was intended primarily as a descriptive real-world evaluation rather than a formal comparative analysis. Finally, the study was not designed to evaluate long-term survival trends or causal associations between TPO-RA exposure and survival outcomes. Nevertheless, we believe that these real-world observations provide clinically relevant insight into evolving patterns of TPO-RA use in pediatric hematology.

### 3.7. Future Perspectives

The future development of TPO-RAs as a class of drugs is inherently linked to ITP, with their use progressively shifting toward earlier and broader integration into treatment strategies. Initial data from adult studies support their potential use in combination with corticosteroids as part of first-line regimens [106,107]. More transformative insights may come from the recent pediatric PINES study, in which eltrombopag demonstrated higher platelet response rates compared with standard first-line therapy in children with newly diagnosed ITP without severe bleeding [20]. In the near future, individualized treatment approaches will likely be of key importance, particularly those integrating TPO-RAs with immunosuppressive therapy in a rational and timely manner. Future prospective studies should also incorporate patient-reported outcomes and standardized quality-of-life measures to better define the broader clinical impact of TPO-RA therapy in pediatric populations.

Further studies are essential to clearly define the role of TPO-RAs in pediatric SAA. Until additional evidence becomes available, their use will likely continue to depend on institutional practices, patient-specific factors, and the complexity of therapeutic decision-making. Future investigation of newer TPO-RAs, which may exert broader effects on early hematopoiesis, may further expand therapeutic options in this challenging condition.

TPO-RAs are also likely to be increasingly adopted in the management of PGF, given their favorable safety profile compared with alternative therapeutic options and the encouraging results reported to date. However, it remains unclear whether TPO-RAs alone are sufficient to achieve complete and durable hematologic recovery in PGF. These questions require further studies to be adequately addressed.

Given their rarity and marked clinical heterogeneity, the use of TPO-RAs in IPDs will likely continue to rely on patient-specific considerations. Nevertheless, the accumulating body of evidence supports the pragmatic use of these agents in patients with clinically significant bleeding or when alternative therapeutic options are limited, as reflected in the algorithm proposed by Zaninetti et al. [103]. In the context of WAS, TPO-RAs are expected to remain primarily a bridging strategy to definitive treatment. However, in selected patients lacking a suitable donor or with contraindications to transplantation, longer-term use may also be considered.

An emerging and conceptually distinct application of TPO-RAs is their use in chemotherapy-induced thrombocytopenia (CIT), which was not reported in our cohort. Early attempts to evaluate their use for the prophylaxis of CIT were largely abandoned due to concerns regarding unnecessary drug exposure. Consequently, current research has shifted toward the use of TPO-RAs in the management of persistent CIT from the beginning of the chemotherapy cycle [108]. While available pediatric studies suggest that TPO-RAs may effectively increase platelet counts, their impact on clinically meaningful endpoints, such as chemotherapy dose intensity and treatment delays, remains inconsistent [109,110,111].

Current recommendations from the International Society on Thrombosis and Haemostasis regarding CIT emphasize enrollment in clinical trials whenever possible. In the off-label setting, romiplostim may be considered in selected patients with solid tumors. In contrast, the use of TPO-RAs in CIT associated with hematologic malignancies remains controversial, primarily due to concerns regarding potential stimulation of malignant clones [112]. Nevertheless, with the growing body of clinical data, broader implementation of TPO-RAs in this indication can be anticipated in the future.

## 4. Materials and Methods

### 4.1. Study Design and Patient Identification

We performed a retrospective single-center analysis of the medical records of patients hospitalized at the Department of Pediatrics, Oncology, and Hematology, Medical University of Lodz, Poland, between 1 January 2016, and 31 March 2026, to identify all cases of TPO-RA use for any hematologic indication. Patients were eligible for inclusion if they received at least one dose of any TPO-RA agent during the study period. No restriction regarding the underlying diagnosis was applied. A total of 35 patients fulfilling the eligibility criteria were identified and included in the final analysis.

### 4.2. Study Population and Collected Variables

Demographic, clinical, treatment, and outcome-related data were extracted retrospectively from routine medical documentation. Collected variables included year of TPO-RA initiation, sex, primary diagnosis, age at diagnosis, age at TPO-RA initiation, indication for TPO-RA therapy, interval from diagnosis to treatment initiation, rescue use status, follow-up duration, response measures (according to the predefined criteria detailed in Section 4.3), treatment modifications, bleeding events during therapy, AEs (assessed as described in Section 4.4), and follow-up status at the last available clinical contact.

Rescue TPO-RA initiation was defined as treatment started because of clinically significant bleeding or refractory thrombocytopenia requiring rapid platelet increase after failure or anticipated insufficient efficacy of previous immunosuppressive therapy.

Follow-up duration was calculated from TPO-RA initiation to the last available clinical assessment or the predefined study data cutoff.

### 4.3. Treatment Exposure Definitions and Response Assessment

Treatment-related analyses included evaluation of the initially administered TPO-RA agent (eltrombopag, romiplostim, or avatrombopag), its efficacy, treatment status, and reasons for therapy discontinuation or modification, including switches between TPO-RAs. Analogous efficacy- and exposure-related variables were assessed for subsequent TPO-RA therapy, when applicable. In the context of overall TPO-RA therapy, treatment status, reasons for therapy discontinuation, and bleeding events occurring during treatment were additionally evaluated.

Treatment efficacy was assessed using predefined platelet response criteria. Initial response (IR) was defined as achievement of platelet count (PLT) > 50 × 10^9^/L in at least one measurement. Complete response (CR) was defined as achievement of PLT > 100 × 10^9^/L in at least one measurement in the absence of clinically relevant bleeding. Sustained response (SR) was defined as PLT > 50 × 10^9^/L maintained during at least 75% of follow-up visits over a 6-month observation period, reflecting approaches used in previous clinical studies [13,18,19]. Assessment of SR required a minimum of 6 months of available follow-up after TPO-RA initiation. Patients not meeting this criterion were classified as non-evaluable for SR analysis rather than as non-responders.

### 4.4. Safety Assessment

Safety analyses included all documented treatment-related AEs identified through retrospective review of routine clinical documentation. The assessment focused particularly on liver toxicity, thromboembolic complications, QTc prolongation or other cardiac adverse effects, bone marrow fibrosis, hypersensitivity reactions, respiratory symptoms, gastrointestinal toxicity, and other clinically relevant toxicities.

Liver toxicity was evaluated using regularly monitored laboratory parameters, including alanine aminotransferase (ALT), aspartate aminotransferase (AST), total bilirubin, activated partial thromboplastin time (aPTT), international normalized ratio (INR), and serum albumin, assessed at least every 3 months or more frequently when clinically indicated. Hematologic abnormalities were evaluated using complete blood count analyses performed during routine follow-up visits. Other AEs were recorded when clinically suspected and documented rather than through protocol-driven active surveillance. Bone marrow fibrosis assessment was based on clinically indicated marrow examinations and was not performed systematically. All events were graded according to the Common Terminology Criteria for Adverse Events (CTCAE), version 5.0 (National Cancer Institute, Bethesda, MD, USA) [27].

### 4.5. Laboratory and Clinical Data Sources

Clinical and laboratory data used for treatment evaluation were obtained from routinely collected medical records and institutional diagnostic testing performed according to standard diagnostic practice. Complete blood counts, liver function parameters, and other hematologic measurements were derived from conventional clinical laboratory analyses used in routine patient care throughout the study period. Given the retrospective design and prolonged observation interval, minor temporal variation in laboratory instrumentation, analytical platforms, or diagnostic workflows cannot be completely excluded and represents a potential source of methodological variability.

### 4.6. Statistical Analysis

Statistical analyses were performed using Statistica 13.1 PL (StatSoft, Cracow, Poland).

Distribution normality of continuous variables was assessed using the Shapiro–Wilk test. Depending on distribution characteristics, continuous variables are presented either as means with standard deviations (SDs) for normally distributed data or as medians with interquartile ranges (IQRs) for non-normally distributed variables. Categorical variables are expressed as absolute numbers and percentages.

Given the retrospective design, limited sample size, and marked clinical heterogeneity of the cohort, the study was designed primarily as a descriptive real-world assessment rather than a formal inferential comparative evaluation.

Nevertheless, exploratory comparative analyses of treatment outcomes among first-line TPO-RA agents were additionally performed in patients with ITP. Comparative analyses were restricted to this subgroup because, in the remaining indications, treatment selection showed a clear preference toward specific TPO-RA agents, precluding meaningful inter-agent comparisons. Associations between categorical treatment outcomes and the initially administered TPO-RA agent were evaluated using the chi-square (χ^2^) test or Fisher’s exact test, when appropriate. Owing to small subgroup sizes and limited statistical power, these analyses should be interpreted cautiously and regarded as exploratory rather than confirmatory. A two-sided *p* value < 0.05 was considered statistically significant.

Analyses were based on available data extracted from medical records. No formal imputation procedures were applied for missing or non-evaluable observations.

### 4.7. Ethical Considerations

The study was approved by the Local Ethical Committee at the Medical University of Lodz (decision no. RNN/261/23/KE issued on 14 November 2023). All patient data were pseudonymized before analysis.

## 5. Conclusions

Our real-world data confirm the high efficacy and favorable safety profile of TPO-RAs in pediatric patients and demonstrate their expanding use beyond traditional ITP indications. Over the past decade, clinical practice at our center has shifted toward broader and, in selected cases, earlier incorporation of TPO-RAs into pediatric thrombocytopenia management. Given the heterogeneity of indications and the limitations of retrospective data, prospective studies are required to better define optimal timing, patient selection, and long-term outcomes, particularly in non-ITP settings.

## Figures and Tables

**Table 1:** Baseline demographic and clinical characteristics of the study cohort.

Characteristic		*n* (%)
**Baseline characteristics**		
Total patients		35
Year of TPO-RA initiation	2016–2020	5/35 (14%)
	2021–2023	10/35 (29%)
	2024–2026	20/35 (57%)
Sex	Male	19/35 (54%)
	Female	16/35 (46%)
Diagnosis	Immune thrombocytopenia	27/35 (77%)
	Severe aplastic anemia	5/35 (14%)
	Poor graft function	2/35 (6%)
	Inherited platelet disorders	1/35 (3%)
Age at diagnosis, median years (IQR)		10.36 (5.49–13.89)
Age at TPO-RA initiation, median years (IQR)		10.78 (6.8–14.65)
Time from diagnosis to TPO-RA start, median days (IQR)		231 (90–424)
Follow-up duration, median days (IQR)		621 (287–1005)
Rescue TPO-RA initiation		8/35 (23%)
**First TPO-RA agent treatment outcome**		
Initial TPO-RA agent	Eltrombopag	22/35 (63%)
	Romiplostim	12/35 (34%)
	Avatrombopag	1/35 (3%)
IR1	Total	27/35 (77%)
	Eltrombopag	14/22 (64%)
	Romiplostim	12/12 (100%)
	Avatrombopag	1/1 (100%)
Time to IR1, median days (IQR)		7 (7–12.5)
CR1	Total	22/35 (63%)
	Eltrombopag	10/22 (45%)
	Romiplostim	11/12 (92%)
	Avatrombopag	1/1 (100%)
SR1 *	Total	19/31 (61%)
	Eltrombopag	12/21 (57%)
	Romiplostim	6/9 (67%)
	Avatrombopag	1/1 (100%)
First TPO-RA therapy status **	Ongoing	14/34 (41%)
	Discontinued	14/34 (41%)
	Switched	6/34 (18%)
Reason for first TPO-RA therapy discontinuation	Disease remission	6/14 (43%)
	HSCT	4/14 (29%)
	Lack of response	2/14 (14%)
	Death	1/14 (7%)
	Transition to hospice care	1/14 (7%)
Reason for first TPO-RA therapy switch	Lack of response	4/6 (67%)
	Dose titration difficulty	1/6 (16.5%)
	Drug unavailability	1/6 (16.5%)
**Overall TPO-RA treatment outcome**		
Overall TPO-RA therapy status **	Ongoing	20/34 (59%)
	Discontinued	14/34 (41%)
Reason for overall TPO-RA therapy discontinuation	Disease remission	5/14 (36%)
	HSCT	4/14 (29%)
	Lack of response	3/14 (21%)
	Death	1/14 (7%)
	Transition to hospice care	1/14 (7%)
Bleeding events during TPO-RA treatment	Any bleeding	7/35 (20%)
	Epistaxis	4/7 (57%)
	Mucosal bleeding	2/7 (29%)
	Menorrhagia	1/7 (14%)

* SR1 assessment was not feasible in four cases due to insufficient follow-up duration. ** Treatment status could not be determined in one patient due to loss to follow-up. Abbreviations: TPO-RA, thrombopoietin receptor agonist; IQR, interquartile range; IR1, initial response to the first TPO-RA; CR1, complete response to the first TPO-RA; SR1, sustained response to the first TPO-RA; HSCT, hematopoietic stem cell transplantation. Detailed definitions of response points are provided in the Section 4 of the manuscript. Detailed patient-level data are presented in the corresponding columns of Supplementary Table S1.

**Table 2:** Treatment-related adverse events observed during TPO-RA therapy.

Adverse Event	*n* (%)	Comments
Any adverse event	9/35 (26%)	All events were graded 1–2 according to CTCAE v5.0
Liver dysfunction	4/35 (11%)	Three cases of hypertransaminasemia and one case of cholestasis; all considered related to eltrombopag therapy
Thrombotic events	0/35 (0%)	-
Cardiac events	0/35 (0%)	-
Bone marrow fibrosis	1/35 (3%)	Moderate reticulin fibrosis (grade 2)
Hypersensitivity	1/35 (3%)	Mild cutaneous lesions, probably related to TPO-RA therapy
Hematologic abnormalities	4/35 (11%)	Mainly anemia, leukocytosis, and eosinophilia, mostly associated with eltrombopag therapy
Other	0/35 (0%)	-

Abbreviations: TPO-RA, thrombopoietin receptor agonist.

**Table 3:** Treatment outcomes and switching patterns of TPO-RAs in patients with ITP.

Characteristic		*n* (%)
Total patients		27
Initial TPO-RA agent	Eltrombopag	15/27 (55%)
	Romiplostim	11/27 (41%)
	Avatrombopag	1/27 (4%)
IR1	Total	23/27 (85%)
	Eltrombopag	11/15 (73%)
	Romiplostim	11/11 (100%)
	Avatrombopag	1/1 (100%)
CR1	Total	19/27 (70%)
	Eltrombopag	8/15 (53%)
	Romiplostim	10/11 (91%)
	Avatrombopag	1/1 (100%)
SR1 *	Total	17/24 (71%)
	Eltrombopag	11/15 (73%)
	Romiplostim	5/8 (63%)
	Avatrombopag	1/1 (100%)
TPO-RA switch	Total	6/27 (22%)
	Eltrombopag → romiplostim	3/6 (50%)
	Romiplostim → eltrombopag	2/6 (33%)
	Avatrombopag → eltrombopag	1/6 (17%)
IR2	Total	2/6 (33%)
	Eltrombopag	2/3 (67%)
	Romiplostim	0/3 (0%)
CR2	Total	2/6 (33%)
	Eltrombopag	2/3 (67%)
	Romiplostim	0/3 (0%)
SR2	Total	1/6 (17%)
	Eltrombopag	1/3 (33%)
	Romiplostim	0/3 (0%)

* SR1 assessment was not feasible in three cases due to insufficient follow-up duration. Abbreviations: TPO-RA, thrombopoietin receptor agonist; ITP, immune thrombocytopenia; IR2, initial response to the second TPO-RA; CR2, complete response to the second TPO-RA; SR2, sustained response to the second TPO-RA. Detailed definitions of response points are provided in the Section 4 of the manuscript.

**Table 4:** Overview of key properties and regulatory status of TPO-RAs [16,17,31,36,37,38,50,55,56,57].

Feature	Eltrombopag	Romiplostim	Avatrombopag	Lusutrombopag	Hetrombopag
Biochemical structure	small non-peptide molecule	peptibody	small non-peptide molecule	small non-peptide molecule	small non-peptide molecule
Route of administration	oral	subcutaneous	oral	oral	oral
Binding site on receptor	transmembrane domain	extracellular domain	transmembrane domain	transmembrane domain	transmembrane domain
Mechanism of receptor activation	non-competitive agonism	TPO-mimetic agonism (competitive)	non-competitive agonism	non-competitive agonism	non-competitive agonism
Key off-target effects	iron and calcium chelation; HSPCs survival effect; DNA damage response activation	immunomodulatory effect (Fc-mediated and Treg-related)	no well-defined off-target effects	no well-defined off-target effects	emerging effects on HSPCs proliferation; off-target mechanisms incompletely defined
Effects on MKs	acts on early progenitors and immature MKs	mainly acts on mature MKs	no clear stage specificity	supports megakaryopoiesis from progenitors (limited data)	supports megakaryopoiesis from progenitors (limited data)
Food-drug interaction	yes—chelation with polyvalent cations; must separate from e.g., dairy, antacids	no food restrictions	no food restrictions	no food restrictions	no food restrictions
Approved indications—FDA (indication and year)	chronic ITP in adults—2008; thrombocytopenia and chronic hepatitis before and during interferon-based therapy—2012; severe aplastic anemia with insufficient response to IST—2014; chronic ITP in children—2015; first-line treatment in combination with IST in SAA in adults and children—2018	chronic ITP in adults—2008; chronic ITP in children—2018; newly diagnosed refractory ITP in adults—2019	before medical procedures in adults with thrombocytopenia and CLD—2018; chronic ITP in adults—2019; chronic ITP in children—2025	before medical procedures in adults with thrombocytopenia and CLD—2018	not approved

Abbreviations: TPO-RAs, thrombopoietin receptor agonists; TPO, thrombopoietin; HSPCs, hematopoietic stem and progenitor cells; MKs, megakaryocytes; FDA, Food and Drug Administration; ITP, immune thrombocytopenia; IST, immunosuppressive therapy; CLD, chronic liver disease.

## Data Availability

Data are available upon request to szymon.janczar@umed.lodz.pl.

## Supplementary Materials

The following supporting information can be downloaded at: https://www.mdpi.com/article/10.3390/ijms27125175/s1.
